# Timeliness of childhood vaccination in Iran: Survival analysis of a national immunization survey, 2019

**DOI:** 10.1016/j.ijregi.2026.101031

**Published:** 2026-09-30

**Authors:** Shahrokh Izadi, Marjan Mansourian, Seyed Mohsen Zahraei, Sahar Seddiq

**Affiliations:** 1 Department of Epidemiology and Biostatistics, School of Health, Isfahan University of Medical Sciences, Isfahan, Iran; 2 CREB, Universitat Politècnica de Catalunya BarcelonaTech (UPC), Barcelona, Spain; 3 Center for Communicable Diseases Control, Ministry of Health and Medical Education, Tehran, Iran

**Keywords:** Vaccine timeliness, Childhood immunization, Survival analysis, Delayed vaccination, National immunization survey Iran, Vaccination coverage

## Abstract

**Objectives:**

Despite high vaccination coverage in Iran, delays in administering age-appropriate vaccines may compromise overall protection. This study used survival analysis to examine the timeliness of vaccine delivery and its sociodemographic correlates.

**Methods:**

We analyzed the vaccination histories of 8248 children aged 24-35 months from the 2019 National Immunization Coverage Survey. Timeliness for the third pentavalent dose (Penta3) and the first measles-mumps-rubella dose (MMR1) was assessed via Kaplan–Meier curves and Cox proportional hazards models, with time-dependent effects where the proportionality assumption was violated. The Gini index was accounted for through stratification.

**Results:**

Although Penta3 coverage was high at 99.13%, only 72.23% of participants had received their vaccinations within the recommended time frame (*P* < 0.001). In contrast, the disparity between total coverage (99.04%) and valid coverage (94.36%) for MMR1 was notably less pronounced (*P* = 0.003). For Penta3, maternal literacy was associated with higher on-time vaccination (hazard ratio [HR] = 1.38; 95% confidence interval [CI] = 1.16-1.66), whereas a birth order ≥4 was associated with lower timeliness (HR = 0.72; 0.63-0.82). For MMR1, urban residence was associated with a lower baseline hazard of on-time vaccination (HR = 0.57; 0.49-0.67), alongside a modest increase in the time-dependent HR (TD-HR = 1.02; 1.01-1.03), suggesting partial convergence over time. Maternal literacy showed no strong main effect for MMR1 (HR = 0.94; 0.64-1.37), with a slight positive time trend (TD-HR = 1.01; 0.99-1.04).

**Conclusion:**

Even with high coverage, gaps in on-time vaccination remain—particularly, among higher-parity children and in urban settings at baseline. The findings underscore the value of routinely reporting timeliness (not just total coverage) and of targeting context-specific barriers (e.g. for large families and urban clinics) to narrow disparities in on-time immunization.

## Highlights

• High vaccination coverage can hide delays in childhood vaccination.
• Third pentavalent dose had the largest gap in valid coverage.
• First measles, mumps, and rubella vaccine dose had smaller validity gaps.
• Higher birth order was linked to delayed childhood vaccination.
• Timeliness monitoring reveals gaps missed by coverage estimates.

## Introduction

Childhood immunization is among the most impactful and cost-effective public health interventions, preventing an estimated 3-5 million deaths annually and substantially reducing the global burden of vaccine-preventable diseases [1]. In recent decades, national immunization programs, particularly, in countries such as Iran, have maintained high coverage, improving child survival and demonstrating the broad health and economic benefits of vaccination [2,3]. Traditionally, program performance has relied on overall coverage; however, evidence shows that coverage alone can mask gaps in on-schedule vaccination. Increasingly, vaccination timeliness—the receipt of each dose within the recommended age window—serves as a more sensitive and epidemiologically meaningful indicator because maximal protection requires on-schedule dosing [[4], [5], [6]].

Delayed vaccination—even when the series is ultimately completed—and missed age-appropriate doses leave children unprotected during critical windows of susceptibility and have been linked to measles outbreaks, greater morbidity requiring hospitalization, and higher mortality; optimal protection requires on-schedule dosing [[7], [8], [9]]. The largest sustained decline in childhood vaccination coverage in nearly three decades has been reported by the World Health Organization (WHO) and the United Nations Children’s Fund, with global coverage with the third dose of diphtheria-tetanus-pertussis–containing vaccine (DTP3) dropping from 86% in 2019 to 81% in 2021 because of COVID-19–related disruptions and other systemic factors [10,11]. Despite near-universal coverage in some settings, delays and missed age-appropriate vaccinations remain a pressing challenge, driven by a complex interplay of demographic, socioeconomic, geographic, and health system factors—including maternal education, birth order, rural–urban residence, accessibility of health services, and parental trust in health care providers [4,6,[12], [13], [14], [15]].

In Iran, several studies have previously examined delayed vaccination and predictors of vaccination timing; however, routine national reporting and many program evaluations have primarily emphasized overall coverage indicators rather than dose-specific timeliness using time-to-event approaches. Therefore, further analysis of nationally representative data can help clarify the extent to which high coverage coincides with on-schedule vaccination [3,[16], [17], [18]]. Evidence from other low- and middle-income countries indicates that examining vaccination timeliness can uncover inequities and systemic gaps that remain hidden when relying solely on coverage estimates, even in settings with ostensibly high coverage [19]. Most studies have relied on cross-sectional designs and conventional coverage indicators, which fail to capture early, on-time, and delayed vaccinations, thereby overlooking important quality dimensions of immunization service delivery [4,6]. Moreover, advanced statistical methods, such as survival analysis, can characterize the temporal distribution of vaccination and identify predictors of delay [20,21]. By contrast, survival analysis techniques, including Kaplan–Meier estimation and Cox proportional hazards models, allow precise, time-sensitive evaluation by accounting for censored data; estimating the rate of receiving age-appropriate vaccination; and assessing associations with individual-, household-, and system-level factors. International applications of these methods have yielded critical insights into timeliness patterns for vaccines such as pentavalent (containing immunizing agents against diphtheria, tetanus, pertussis, hepatitis B, and *Haemophilus influenzae* type b) and measles-mumps-rubella (MMR) across diverse contexts [4,6,9,12,14].

Recent evidence from Brazil further illustrates the importance of moving beyond crude coverage indicators. In the Brazilian National Vaccination Coverage Survey 2020, which evaluated children born in 2017-2018, complete vaccination coverage and valid dose coverage varied substantially across regions and population groups, and lower coverage was observed among socioeconomically disadvantaged families. These findings support the need to evaluate not only whether vaccines were eventually received but also whether they were administered at valid ages and within recommended time windows [22,23]. A valid (age-appropriate) dose was defined according to the vaccine-specific age window and, where applicable, the minimum inter-dose interval specified in the Iranian National Immunization Guidelines [24]. For the vaccines examined here, doses administered more than 30 days after the recommended target age were classified as delayed; the detailed vaccine-specific criteria are provided in the subsequent section.

This study applies survival analysis to nationally representative 2019 data from Iran’s Ministry of Health coverage survey [3] and, focusing on Pentavalent-3 (Penta3) (the third dose of pentavalent vaccine at the age of 6 months) and MMR1 (the first dose of MMR at the first year of life) in children aged 24-35 months, uses established epidemiologic definitions of age-appropriate vaccination and time-to-event methods [17,18] to generate actionable insights for improving program effectiveness, efficiency, and equity.

Penta3 and MMR1 were selected because they represent complementary dimensions of vaccination timeliness. Penta3 reflects cumulative adherence to a multidose primary vaccination schedule because its validity depends on the appropriate receipt and spacing of preceding doses, whereas MMR1 represents a key age-specific vaccination milestone around the first birthday. Together, these vaccines, therefore, provide informative but distinct indicators of schedule adherence within the first year of life.

## Methods

### Study design and population

This study used data from a nationally representative cross-sectional survey of children aged 24-35 months, conducted in the summer of 2019 by the Ministry of Health and Medical Education of the Islamic Republic of Iran [3]. The precise birth dates and vaccine administration dates were extracted from the children’s vaccination cards, which serve as official health records documenting all significant health events during the first 12 years of life and are retained by families. In addition, all records are stored in a web-based Integrated Comprehensive Health Information System (ICHIS), known as SIB in Persian [25], which ensures that, in cases where the physical vaccination card was unavailable, data could be retrieved. Consequently, all pertinent dates and data necessary for the analysis were fully documented for each participant, ensuring that no individuals were excluded from the study because of incomplete information. The sociodemographic data were collected through structured face-to-face interviews with parents or legal guardians using the same questionnaire used to collect vaccination information. Fieldwork for this survey was conducted from June 15 to July 25, 2019. Of the 8682 children surveyed, 8248 (95.0%) were Iranian, whereas 434 (5.0%) were non-Iranian. This study included only participants from Iran. Comprehensive details regarding the fieldwork implementation, the questionnaire used, and the sampling methodology have been documented in previous publications [3].

The sampling frame has been described in detail elsewhere [3]. Briefly, using the 2016 Population and Housing Census from the Statistical Center of Iran, the sample was allocated across all 31 provinces in proportion to provincial population size. A total of 1447 clusters were selected using probability-proportional-to-size sampling, with six eligible children sampled per cluster, yielding 8682 children in the original survey. Within selected population centers, households were identified through the local health service structure and systematic or random sampling procedures. When more than one eligible child resided in a household, information was collected for the youngest eligible child. Of the 8682 surveyed children, 434 were non-Iranian and were excluded from the present analysis, leaving 8248 Iranian children. Because exclusion was performed at the individual level, the number of Iranian children retained per original cluster varied from one to six. The original cluster identifiers were retained in the analysis to account for the clustered sampling design. This cluster-based sampling approach was consistent with WHO recommendations for immunization coverage surveys [26].

The original survey used a cross-sectional cluster sampling design [3]; however, the availability of exact dates of birth, interview dates, and vaccine administration dates allowed individual vaccination histories to be reconstructed retrospectively. Vaccination dates were obtained from the child’s immunization card and, when needed, verified through the web-based ICHIS (known as SIB in Persian) [25]. Therefore, the present analysis was conducted as a retrospective time-to-event analysis reconstructed from documented vaccination histories rather than as a simple cross-sectional comparison of crude coverage. For each vaccine, children entered the risk set at the vaccine-specific age of eligibility and were followed up until receipt of a valid age-appropriate dose or censoring, according to the vaccine-specific rules described in the subsequent section.

Vaccine age appropriateness was defined according to the 2024 National Immunization Guidelines of Iran [24]. Detailed vaccine-specific definitions are described in subsequent sections.

### Outcome definition

The primary event of interest was receipt of an age-appropriate (valid) vaccine dose, defined as administration within the nationally recommended age window and meeting the minimum inter-dose interval criteria.

Children who received a valid dose during the observation period were coded as events (event = 1). Those who had not received the vaccine by the interview date were treated as right-censored at their age at interview.

Doses administered outside the valid window (either too early or too late) were considered invalid vaccinations. These cases were excluded from the definition of “valid” events but remained in the risk set until their date of vaccination, ensuring proper handling of their person-time.

### Vaccination-specific analytical procedures

For each vaccine, the analytic time origin was defined as the earliest age at which a child became eligible to receive a valid dose, as stipulated in the Iranian Ministry of Health’s National Immunization Guideline. Establishing time zero in this manner minimizes the likelihood of misclassifying unexposed person-time as exposed, thereby enabling unbiased estimation of rates.

Given the variability in eligibility periods and minimum age requirements across vaccines, each was analyzed independently by applying vaccine-specific definitions for survival time, censoring criteria, and inclusion parameters, as described in the subsequent section.

### Pentavalent dose 3

The prerequisite for the validity of the Penta3 is receiving the first and second doses of the pentavalent vaccine at the appropriate age. The proper age for the first dose of the pentavalent vaccine was defined as administration at ≥42 days of age (6 weeks), and, for the second dose, it was required to be administered ≥30 days after Penta1. Penta3 age appropriateness required ≥60 days from the second dose and ≥120 days from the first dose.

For each participant meeting the criteria for an age-appropriate Penta3, follow-up was defined as starting on the date of the second pentavalent dose. Administration of the Penta3 beyond 210 days of age (i.e. 6 months + 30 days) was classified as delayed and was treated as censored.

### MMR1

The history of the first shot of the MMR was considered valid (age-appropriate) if administered at ≥358 days of age (i.e. 12 months-7 days). According to the last version of the Immunization Guidelines [24], this vaccine should be administered on the first birthday; however, for the sake of accommodating weekly clinic scheduling and minimizing vial wastage, those administering this vaccine usually do so on only one of the days of the week, so the first week before the first birthday was added to the valid time span. Thus, shots before 358 days were considered age-inappropriate, and those given >30 days after the first birthday (i.e. ≥396 days) were classified as delayed and were treated as censored.

### Statistical analysis

Descriptive analyses were performed to summarize the distribution of valid (age-appropriate) vaccinations for the Penta3 and the first dose of the MMR1 vaccine across key sociodemographic characteristics, including the child’s sex, residence (urban/rural), maternal education (literate/illiterate), birth order (first to third vs ≥fourth child), and the provincial Gini coefficient (categorized as 0.25 to <0.30, 0.30 to <0.35, 0.35 to <0.40, and 0.40 to <0.45). The Gini coefficient is an ecological measure of economic inequality; values closer to zero indicate less inequality. Kaplan–Meier survival curves were constructed to visualize the cumulative probability of receiving an age-appropriate vaccine dose.

Survival time was defined from each child’s age at eligibility for the respective vaccine until receipt of a valid dose or censoring at the date of interview. This design ensured that participants contributed person-time only after becoming eligible for vaccination. The associations between sociodemographic variables and the timing of vaccine uptake were examined using Cox proportional hazards (PHs) models. The PHs assumption was evaluated using Schoenfeld residuals; variables that showed violations were modeled as time-dependent covariates.

Primary covariates included birth order, maternal education, and area of residence. The provincial Gini coefficient, obtained from the Statistical Center of Iran (2019) [27], was treated as a stratification factor because it violated the PHs assumption and was measured at an ecological (provincial) level. As such, hazard ratios (HRs) were not estimated for this variable. A Z-test was used for comparisons of means and proportions in descriptive analyses.

Because the original survey used a probability-proportional-to-size cluster sampling design, the analysis was conducted with attention to the clustered nature of the data. Cluster-level dependence was accounted for using robust sandwich variance estimation clustered at the primary sampling unit. Because the sampling design was approximately self-weight and no separate individual-level sampling weight variable was available in the analytic data set, weighted estimates were not used. This issue was also considered when interpreting the generalizability of the findings.

All statistical analyses were conducted using Stata version 17 (StataCorp LLC, College Station, TX, USA). Two-sided *P* < 0.05 were considered statistically significant.

## Results

Among 8248 Iranian children aged 24-35 months, the Penta3 total coverage was 99.13%, and valid age-appropriate coverage was 72.23%, a difference of 26.90 percentage points. For MMR1, the total coverage was 99.04% and valid age-appropriate coverage was 94.36%, a difference of 4.68 percentage points.

Valid Penta3 coverage was lower among children of illiterate mothers than among children of literate mothers (62.36% vs 72.58%; log-rank *P* < 0.001) and lower among children with birth order ≥4 than those with birth order 1-3 (61.5% vs 72.9%; log-rank *P* < 0.001). Valid MMR1 coverage was also lower among higher–birth order children (90.1% vs 94.6%; log-rank *P* = 0.014) and among urban children compared with rural children (93.71% vs 96.19%; log-rank *P* < 0.001). Across Gini coefficient strata, valid coverage declined in the highest inequality category for both vaccines.

Figure 1, Figure 2 depict the time to age-appropriate vaccination by maternal education and Gini coefficient, respectively, for the Penta3 and the MMR1.

**Figure 1:**
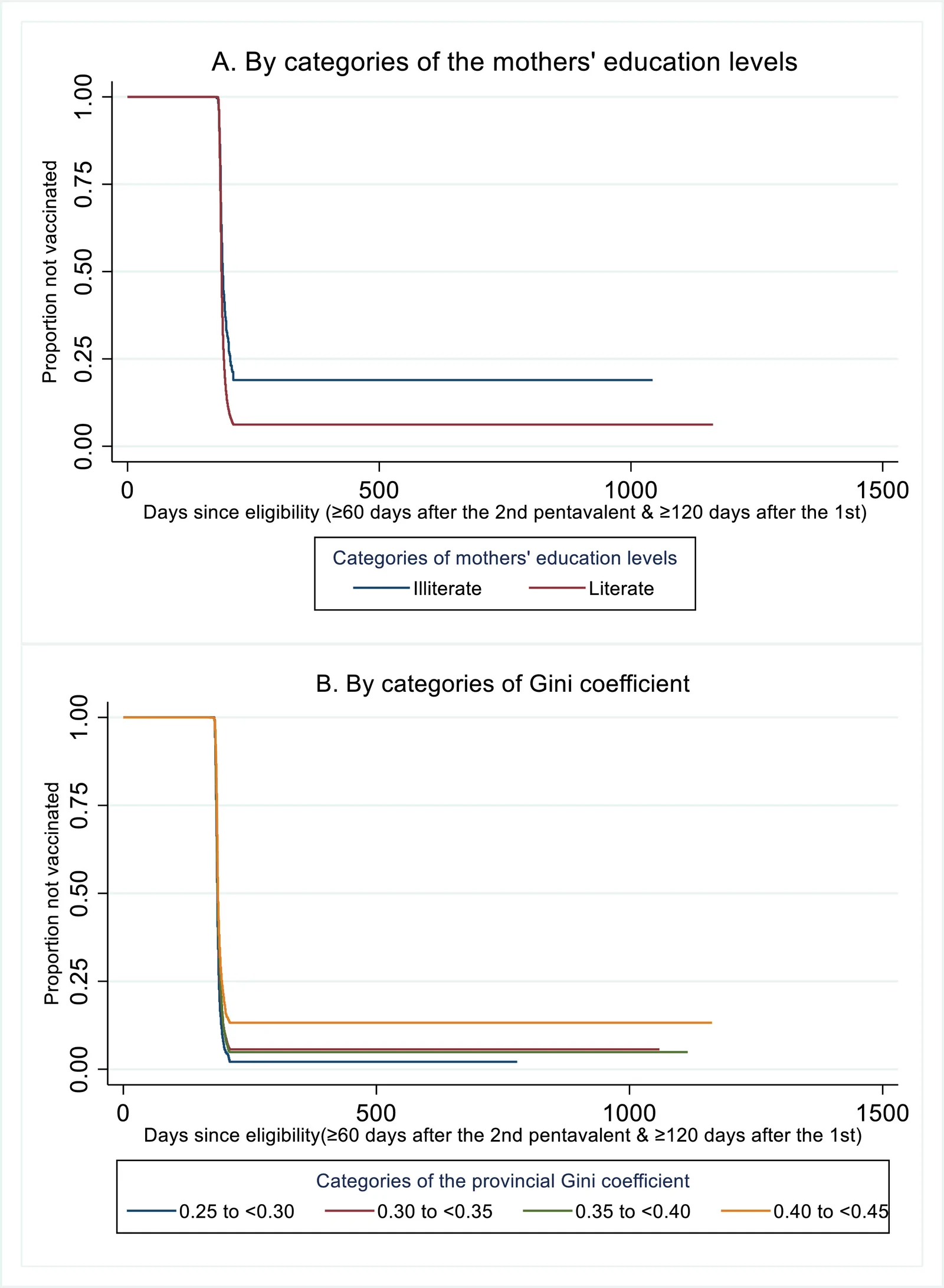
Kaplan–Meier plot of the time to the third pentavalent shot in children aged 24-35 months, by (a) maternal education level and (b) Gini coefficient of the provinces of residence of the children. Vaccination Coverage Survey, Iran, 2019.

**Figure 2:**
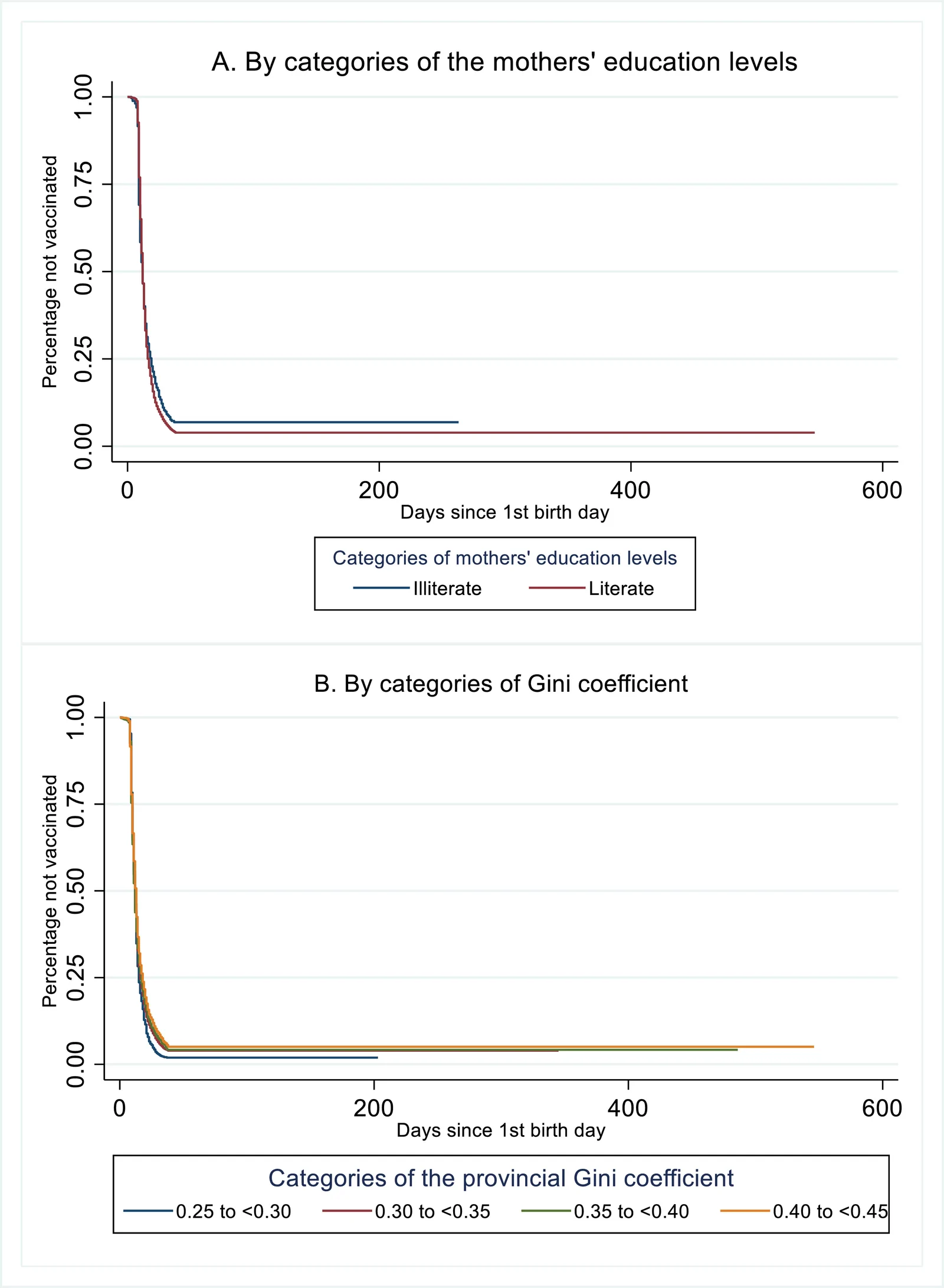
Kaplan–Meier plot of the time to the first measles-mumps-rubella vaccine shot in children aged 24-35 months, by (a) maternal education level and (b) Gini coefficient of the provinces of residence of the children. Vaccination Coverage Survey, Iran, 2019.

In the adjusted Cox regression models, birth order ≥4 was associated with a lower hazard of age-appropriate Penta3 vaccination compared with birth order 1-3 (HR = 0.72; 95% CI = 0.63-0.82). Maternal literacy was associated with a higher hazard of age-appropriate Penta3 vaccination (HR = 1.38; 95% CI = 1.16-1.66). For MMR1, urban residence was associated with a lower baseline hazard of age-appropriate vaccination than rural residence (HR = 0.57; 95% CI = 0.49-0.67), although the positive time-dependent effect suggested partial convergence over time (TD-HR = 1.02; 95% CI = 1.01-1.03). Birth order ≥4 was also associated with lower MMR1 timeliness (HR = 0.86; 95% CI = 0.77-0.96). The provincial Gini coefficient was incorporated as a stratification factor because it violated the PHs assumption and was measured at the ecological level. Figure 3 shows the smoothed hazard estimate by provincial Gini coefficient groups for the Penta3. Even visually, it is evident that the PHs assumption does not hold.

**Figure 3:**
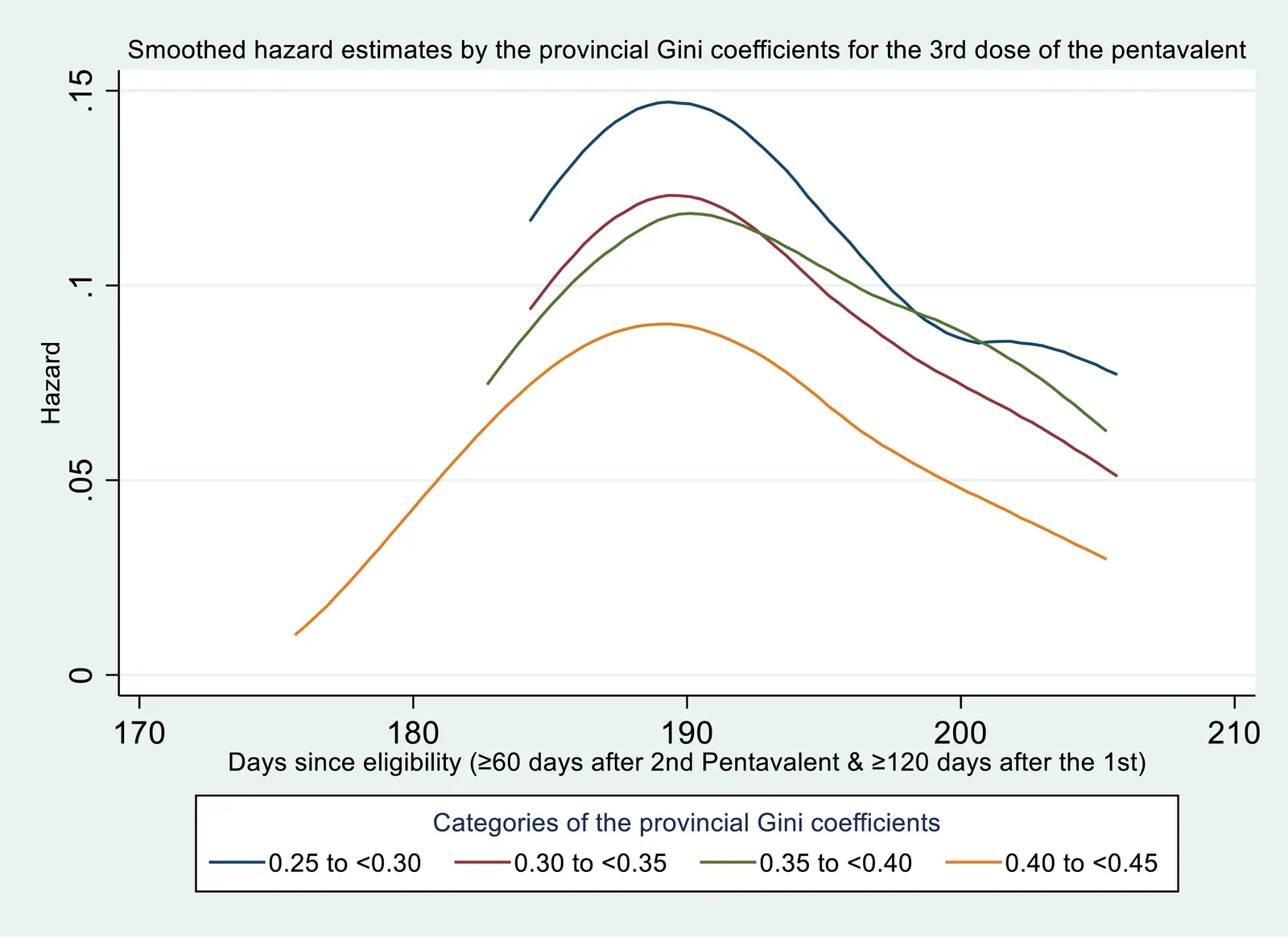
Smoothed hazard estimates by the categories of the provincial Gini coefficient for the third dose of pentavalent, survey of the vaccination coverage of children aged 24-35 months, Islamic Republic of Iran, summer 2019, N = 8248.

## Discussion

This nationally representative analysis showed that high total vaccination coverage in Iran does not necessarily translate into optimal timeliness. The gap was particularly evident for the Penta3, whereas MMR1 showed a smaller difference between total and timely coverage. The findings also indicated that delayed or untimely vaccination was not randomly distributed: higher birth order, maternal illiteracy, urban residence for MMR1, and higher provincial inequality were associated with poorer timeliness patterns. These results highlight the value of incorporating timeliness indicators into routine immunization monitoring, especially in countries where total coverage is already high.

The findings suggest that program performance should be evaluated not only by whether children eventually receive vaccines but also by whether doses are administered at ages that provide appropriate protection. This distinction is particularly important for multidose vaccines, where age eligibility and minimum inter-dose intervals determine validity. Therefore, monitoring age-appropriate vaccination can identify service delivery gaps that remain hidden when only crude coverage is reported.

Findings similar to these have been reported in Saudi Arabia. A cross-sectional study in Sakakah, Al-Jouf (northern Saudi Arabia) showed that children of less-educated mothers and those with higher birth orders were significantly more likely to experience vaccination delays beyond 4 weeks after the recommended schedule [28]. These patterns closely align with our observations and highlight the persistent influence of maternal education and family structure on vaccination timeliness. Comparable patterns have been reported in Nepal, where total coverage exceeded 90% for all vaccines. Yet, age-appropriate coverage fell as low as 41.5% for the Penta3. In similar studies conducted in Oman, despite nearly complete total coverage, only 78% of children had received age-appropriate (i.e. valid) vaccinations [29,30]. Analyses of the US National Immunization Survey highlight schedule quality issues: about 10.5% of children received at least one invalid dose, and excluding invalid doses modestly lowers coverage estimates [31,32]. In addition, multi-country analyses across 45 low- and middle-income settings found that children received the DTP3 dose a median of 6 weeks late, with one-quarter being at least 19 weeks overdue [6]. Studies in Uganda, Senegal, and Nepal highlight maternal literacy and urban residence as consistent predictors of on-time vaccination, with higher birth order and large family size often being associated with delays [9,29,33].

In the present study, the Gini coefficient was determined and reported by province, based on reports from the Statistical Center of Iran [27]. As previously mentioned, Gini values closer to zero indicate less inequality. Looking at Table 1 and the Kaplan–Meier survival curves (Figure 1, Figure 2), the trend of timely vaccine receipt decreases with an increasing Gini coefficient for both vaccines (*P* < 0.001, linear-by-linear test for both vaccines). These findings are broadly consistent with evidence from the United States showing socioeconomic and insurance-related differences in vaccination timeliness [32].

**Table 1:** Valid and total vaccine doses for children (aged 24-35 months) participating in the 2019 vaccination coverage survey (Iran), by demographic characteristics.

Variable	Category	Third pentavalent				First MMR			
		Eligible (N)	Valid^a^ N (%)	Total coverage^b^ N (%)	*P*-value (Log-rank test)	Eligible (N)	Valid^a^ N (%)	Total coverage^b^ N (%)	*P*-value (log-rank test)
Sex	Male	4384	3178 (72.5)	4344 (99.09)	0.955	4384	4138 (94.39)	4337 (98.93)	0.899
	Female	3864	2780 (71.9)	3832 (99.17)		3864	3645 (94.33)	3832 (99.17)	
	Total	8248	5958 (72.23)	8176 (99.13)		8248	7783 (94.36)	8169 (99.04)	
Residence	Urban	6093	4435 (72.8)	6025 (98.88)	0.397	6093	5710 (93.71)	6017 (98.75)	<0.001
	Rural	2155	1523 (70.7)	2152 (99.86)		2155	2073 (96.19)	2152 (99.86)	
	Total	8248	5958 (72.23)	8177 (99.14)		8248	7783 (94.36)	8169 (99.04)	
Mother’s education	Literate	7969	5784 (72.58)	7908 (99.23)	<0.001	7969	7539 (94.6)	7901 (99.15)	0.161
	Illiterate	279	174 (62.36)	269 (96.42)		279	244 (87.5)	268 (96.06)	
	Total	8248	5958 (72.23)	8177 (99.14)		8248	7783 (94.36)	8169 (99.04)	
Birth order	Up to the third	7804	5685 (72.9)	7739 (99.17)	<0.001	7804	7383 (94.6)	7733 (99.09)	0.014
	Fourth or more	444	273 (61.5)	437 (98.42)		444	400 (90.1)	436 (98.20)	
	Total	8248	5958 (72.23)	8176 (99.13)		8248	7783 (94.36)	8169 (99.04)	
Gini index	0.25 to <0.30	780	619 (79.4)	779 (99.9)	<0.001	780	760 (97.44)	779 (99.87)	<0.001
	0.3 to <0.35	4905	3549 (72.4)	4889 (99.7)		4905	4660 (95.01)	4884 (99.57)	
	0.35 to <0.40	989	746 (75.4)	983 (99.4)		989	938 (94.84)	984 (99.49)	
	0.4 to <0.45	1574	1044 (66.3)	1525 (96.9)		1574	1425 (90.53)	1522 (96.70)	
	Total	8248	5958 (72.23)	8176 (99.13)		8248	7783 (94.36)	8169 (99.04)	

a Valid (age-appropriate) dose was defined as a dose administered within the recommended age window and meeting the minimum inter-dose interval criteria.

b Percentages are calculated within each subgroup as follows: for valid, (valid ÷ eligible) × 100; for total coverage, (vaccinated [valid + invalid] ÷ total sample) × 100. Overall totals at the bottom use N = 8248 children. Abbreviation: MMR, measles-mumps-rubella.

In the current analysis, we observed that for MMR1, children living in urban areas had a significantly lower likelihood of receiving timely vaccinations than those from rural regions (HR = 0.57, 95% confidence interval [CI] = 0.49-0.67). Although this urban disadvantage exhibited a slight improvement over time, as reflected in the time-dependent HR (TD-HR = 1.02, 95% CI = 1.01-1.03), the extent of this improvement remained minimal (Table 2). The observed phenomenon can be attributed to the more robust health care network in rural areas. In these regions, each household receives direct oversight from health workers operating out of rural clinics, known as health houses. This contrasts sharply with urban areas, where such a comprehensive structure and supervision are lacking because of insufficient human resources. In addition, it is important to highlight the concerted efforts made before 2019 aimed at eliminating measles and rubella in Iran [34]. These initiatives have significantly enhanced the functionality of the existing health care network in rural settings, allowing improved health outcomes.

**Table 2:** Adjusted HRs and TD-HRs for age-appropriate vaccination with Penta3 and MMR1.

Variable	Category	Penta3 HR (95% CI)	Penta3 TD-HR (95% CI)	MMR1 HR (95% CI)	MMR1 TD-HR (95% CI)
Birth order	≥4th rank = 1 first-third birth order = 0	0.72 (0.63-0.82)	No tvc	0.86 (0.77-0.96)	No tvc
Residence	Urban = 1 vs Rural = 0	not retained	No tvc	0.57 (0.49-0.67)	1.02 (1.01-1.03)
Mother’s education	Literate = 1 Illiterate = 0	1.38 (1.16-1.66)	No tvc	0.94 (0.64-1.37)	1.01 (0.99-1.04)

In the fitted models for both vaccines, the effect of the Gini coefficient was adjusted using stratification. Gini coefficient strata (0.25 to <0.30, 0.30 to <0.35, 0.35 to <0.40, 0.40 to <0.45).

Abbreviations: HRs, hazard ratios; No tvc, no significant time-varying component; MMR, measles-mumps-rubella; Penta3, third pentavalent dose; TD-HRs, time-dependent hazard ratios.

Delays among higher-order children, as seen for Penta3 and MMR1 in the present study, are consistent with evidence from Uganda and multi-country analyses showing that larger family size can reduce parental capacity for timely clinic attendance and contribute to vaccination delays [6,19]. Maternal literacy, which improved timeliness for Penta3 in our data (Table 1), has similarly been shown elsewhere to predict adherence to vaccination schedules [35,36]. Observations in other countries also report an urban advantage for timeliness because of better access and awareness [13].

## Strengths and limitations

A major strength of this study was the use of objective vaccination records rather than parental recall, which reduced the risk of recall bias. The study also used a large, nationally representative data set and applied time-to-event methods to distinguish total vaccination from age-appropriate vaccination. Nevertheless, several limitations should be acknowledged. First, the data were obtained from a cross-sectional survey; although exact vaccination dates allowed retrospective reconstruction of time-to-event histories, causal interpretations remain limited. Second, socioeconomic status was represented only by the provincial Gini coefficient, an ecological measure that may not capture household-level economic conditions. Third, the analytic data set did not include detailed measures of access to services, such as distance to the nearest clinic, transportation barriers, or clinic workload. Fourth, although cluster-level dependence was addressed using robust variance estimation, no separate individual-level sampling weight variable was available for a fully weighted survey analysis. Finally, the probability-proportional-to-size cluster sampling design may have provided less representation for very small villages, remote populations, and nomadic communities. Future studies should incorporate household-level socioeconomic indicators, geospatial measures of service access, and spatial survival modeling to better identify local barriers to timely vaccination.

## Conclusion

Despite high national coverage, important gaps in age-appropriate vaccination remain in Iran’s immunization program. Kaplan–Meier and Cox regression analyses identified differences in vaccination timeliness according to maternal education, birth order, urban–rural residence, and provincial inequality, highlighting that high coverage alone is insufficient without timely delivery. Closing this gap will require strengthening service delivery and empowering caregivers using proven tools such as short message service reminders. Integrating timeliness indicators into national monitoring systems will enhance accountability and equity in immunization performance.

## Funding

The original study implemented in 2019, received financial support from the WHO, Tehran Office, through project registration number 2018/863108-1.

## Ethical considerations

The study protocol—entailing analysis of de-identified secondary survey data with no direct participant contact—was reviewed and approved by the Ethics Committee of Isfahan University of Medical Sciences (Approval Code: IR. MUI. DHMT. REC.1403.064), with consent requirements waived in accordance with national regulations.

## Data availability statement

The data set underpinning this analysis is owned by the Ministry of Health and Medical Education and is subject to data protection regulations. Although the data set is not publicly available due to privacy and legal restrictions, it may be accessed upon the submission of a justified request to and subsequent approval by the Ministry’s Center for Communicable Diseases Control.

## CRediT authorship contribution statement

**Shahrokh Izadi:** Writing – review & editing, Validation, Supervision, Methodology, Conceptualization. **Marjan Mansourian:** Writing – review & editing, Validation, Supervision, Methodology, Formal analysis, Conceptualization. **Seyed Mohsen Zahraei:** Writing – review & editing, Resources, Data curation. **Sahar Seddiq:** Writing – review & editing, Writing – original draft, Visualization, Project administration, Investigation, Formal analysis.

## Declaration of competing interest

The authors have no competing interests to declare.
